# Strategies for managing rival bacterial communities: Lessons from burying beetles

**DOI:** 10.1111/1365-2656.12725

**Published:** 2017-08-21

**Authors:** Ana Duarte, Martin Welch, Chris Swannack, Josef Wagner, Rebecca M. Kilner

**Affiliations:** ^1^ Department of Zoology University of Cambridge Cambridge UK; ^2^ College of Life and Environmental Sciences University of Exeter Cornwall UK; ^3^ Department of Biochemistry University of Cambridge Cambridge UK; ^4^ Pathogen Genetics Programme Wellcome Trust Sanger Institute Hinxton UK

**Keywords:** animal behaviour, antimicrobials, microbiome, social immunity

## Abstract

1. The role of bacteria in animal development, ecology and evolution is increasingly well understood, yet little is known of how animal behaviour affects bacterial communities. Animals that benefit from defending a key resource from microbial competitors are likely to evolve behaviours to control or manipulate the animal's associated external microbiota.
2. We describe four possible mechanisms by which animals could gain a competitive edge by disrupting a rival bacterial community: “weeding,” “seeding,” “replanting” and “preserving.” By combining detailed behavioural observations with molecular and bioinformatic analyses, we then test which of these mechanisms best explains how burying beetles, *Nicrophorus vespilloides*, manipulate the bacterial communities on their carcass breeding resource.
3. Burying beetles are a suitable species to study how animals manage external microbiota because reproduction revolves around a small vertebrate carcass. Parents shave a carcass and apply antimicrobial exudates on its surface, shaping it into an edible nest for their offspring. We compared bacterial communities in mice carcasses that were either fresh, prepared by beetles or unprepared but buried underground for the same length of time. We also analysed bacterial communities in the burying beetle's gut, during and after breeding, to understand whether beetles could be “seeding” the carcass with particular microbes.
4. We show that burying beetles do not “preserve” the carcass by reducing bacterial load, as is commonly supposed. Instead, our results suggest they “seed” the carcass with bacterial groups which are part of the *Nicrophorus* core microbiome. They may also “replant” other bacteria from the carcass gut onto the surface of their carrion nest. Both these processes may lead to the observed increase in bacterial load on the carcass surface in the presence of beetles. Beetles may also “weed” the bacterial community by eliminating some groups of bacteria on the carcass, perhaps through the production of antimicrobials themselves.
5. Whether these alterations to the bacterial community are adaptive from the beetle's perspective, or are simply a by‐product of the way in which the beetles prepare the carcass for reproduction, remains to be determined in future work. In general, our work suggests that animals might use more sophisticated techniques for attacking and disrupting rival microbial communities than is currently appreciated.

## 1 INTRODUCTION

Advances in sequencing technology have revealed the full extent of bacterial diversity living within and alongside animals. Although it is becoming clear that bacteria play important roles in animal development, ecology and evolution (Ezenwa, Gerardo, Inouye, Medina, & Xavier, 2012; McFall‐Ngai et al., 2013), it is less well‐understood how animal behaviour influences bacterial communities. In some instances, even though animals undoubtedly perturb the structure and membership of bacterial communities that exist in the animal's proximity, it is unlikely that these behavioural traits have been selected specifically for this purpose. For example, disturbances caused by soil foraging animals lead to shifts in soil microbial communities (Eldridge et al., 2015); in laboratory populations of *Drosophila* flies, the presence and density of flies changes the microbial communities in the flies’ food (Wong et al., 2015). Here we focus instead on behavioural traits that have evolved to control or manipulate the animal's associated external microbial community, and which are particularly likely to be seen in animals that can gain fitness by defending a key resource from microbial competitors. These traits are more likely to occur in animals that depend on a rapidly decaying resource such as a cadaver or fallen fruit, where there is intense selection to minimize competition with microbes (Janzen, 1977). A similar argument is made by Otti, Tragust, and Feldhaar (2014), who argue that microbial pressure and spatio‐temporal variation in the environment are the main selective forces shaping the evolution of external immune defences. In environments where microbial pressure is high and variation is low, we would expect selection to favour the evolution of external immune defences, such as antimicrobial secretions and hygienic behaviours. Here, we address the effects of such external immune defences on microbial communities in the external environment. We describe four contrasting mechanisms by which animals might limit the threat from microbial rivals. We derive contrasting predictions for these mechanisms as to the effect on microbial communities and test them with a detailed analysis of the way in which one insect species alters a rival bacterial community on its key breeding resource.

We call the first of these mechanisms “preserving.” The suggestion here is that animals produce antimicrobial substances that reduce the number of microbes on the resource, so privatizing it for the animal's exclusive use (Strassmann & Queller, 2014). A range of antimicrobial secretions, such as antimicrobial peptides (AMPs) and lysozymes, have been found in secretions of species such as blowflies, hide beetles and burying beetles (Cotter, Topham, Price, & Kilner, 2010; Degenkolb, Düring, & Vilcinskas, 2011; Kerridge, Lappin‐Scott, & Stevens, 2005). It has been suggested that these antimicrobial substances serve to reduce microbial load on the resource (e.g. Rozen, Engelmoer, & Smiseth, 2008). Another example of “preserving” could be the production of a blend of lactones and isocoumarins by the larvae of the parasitoid wasp *Ampulex compressa*, which acts as a defence against the broad range of microbes that may infest the wasp's host, the cockroach *Periplaneta Americana* (Herzner et al., 2013). Alternatively, animals could use plant products with antimicrobial properties to reduce microbial pressure, as wood ants do when they bring tree resin back to their nests to protect the colony against pathogens (Chapuisat, Oppliger, Magliano, & Christe, 2007).

Rather than decreasing overall microbial numbers, animals may instead manage the microbiota on their resources by inducing shifts in the composition of the bacterial community, favouring beneficial groups and eliminating detrimental ones. Mechanistically, this could occur in three different ways. One of them is “weeding,” which involves the selective removal of members of the microbial community. In contrast to “preserving,” this mechanism may not lead to an overall reduction in bacterial load, because in the absence of the “weeded” groups, other bacteria may proliferate. For this purpose, animals might secrete antimicrobial substances themselves: for example *Tribolium* flour beetles externally secrete benzoquinones which have different levels of activity against different bacterial species and seemingly little or no activity against a set of fungal species (Yezerski, Ciccone, Rozitski, & Volingavage, 2007). A comparison of bacterial communities in flour with and without *Tribolium* beetles suggests the beetles reduce species richness in the environment by preventing some bacteria (*Enterobacter* and *Enterococcus*) from colonizing the flour. Mutualistic bacteria may also be involved in the production of antimicrobial defences that “weed” detrimental microbes. For example, fungus‐growing ants and termites have special glands that secrete antibacterial and antifungal substances to protect their symbiotic fungus from competition from other microbes (Cremer, Armitage, & Schmid‐Hempel, 2007; Do Nascimento, Schoeters, Morgan, Billen, & Stradling, 1996; Rosengaus, Traniello, Lefebvre, & Maxmen, 2004). These ants and termites also harbour mutualistic bacteria that produce compounds which selectively eliminate antagonistic fungi from their fungal gardens (Currie, Scott, Summerbell, & Malloch, 1999; Um, Fraimout, Sapountzis, Oh, & Poulsen, 2013).

The other two mechanisms by which community compositions may be changed involve increasing interference competition between microbes (Scheuring & Yu, 2012) and adding new groups to the existing microbial communities. One such mechanism we call “seeding.” In this mechanism, the animal harnesses the competitive advantages of other microbes (e.g. antibiotic production and biofilm formation) to eliminate major microbial rivals. Importantly, the microbes are carried by the animal itself, in a long‐standing obligate mutualism, and the animal inoculates the contested resource with these symbiotic microbes. An example of seeding is the European beewolf, which harbours symbiotic *Streptomyces* bacteria in specialized antennal glands (Kaltenpoth, Göttler, Herzner, & Strohm, 2005). The beewolf female smears the symbionts on the ceiling of the brood cell. The symbionts prevent fungal and bacterial growth by producing a highly effective cocktail of antibiotics (Kroiss et al., 2010).

A final mechanism, related to the “seeding” mechanism, is “replanting.” Here the animal again harnesses the competitive advantages of other microbes to manipulate the external microbiota, and does not entirely eliminate the microbial community from the contested resource. This time, however, it relocates some existing members of the associated external bacterial community for this purpose. The relocated bacteria out‐compete their new neighbouring microbes and might proliferate themselves on the resource. An example of “replanting” may be found in female medflies, which transfer nitrogen‐fixing and pectinolytic bacteria during oviposition to the fruit in which larvae will develop (Behar, Jurkevitch, & Yuval, 2008). The bacteria proliferate on the fruit, accelerating decay and potentially provide nutritional benefits to the larvae. Although these bacteria are present in the medfly's gut microbiota, they are also commonly found free‐living in plants, suggesting a facultative mutualism between the bacteria and medflies.

“Weeding,” “seeding” and “replanting” are not mutually exclusive mechanisms. Nevertheless, predictions can be derived to identify the mechanism(s) by which animals manage competing bacterial populations. (i) “Preserving” should lead to a reduction in bacterial load in the resource manipulated by the animal in comparison to an unmanipulated resource, whereas this is not necessarily predicted for other mechanisms; (ii) new bacterial groups (unobserved on the resource in the absence of manipulation by the animal) should appear in bacterial communities manipulated by “seeding” or “replanting,” but not by “preserving” or “weeding”; and (iii) if animals are “seeding” their resource with beneficial bacteria, these “seeded” groups should be in the animals’ own microbiota, in a long‐standing evolutionary association.

We investigated which of these mechanisms best accounts for the way in which burying beetles, *Nicrophorus vespilloides*, manage the bacterial community on their breeding resource. Burying beetles prepare small vertebrate carcasses for reproduction by shaving off the fur or feathers, rolling the carcass into a ball, coating it with oral and anal exudates and burying it in the soil where it becomes an edible nest for their larvae (Pukowski, 1933; Scott, 1998). Expression of insect lysozyme is up‐regulated both in the adult beetles’ gut and in the anal exudates during reproduction (Jacobs et al., 2016; Palmer et al., 2016), by which means the beetle could “preserve” or “weed” the carcass. The antibacterial activity of *N. vespilloides* exudates has been demonstrated against lysozyme‐susceptible bacteria (Arce, Johnston, Smiseth, & Rozen, 2012; Cotter et al., 2010). When applying exudates to the carcass, beetles could also potentially be “seeding,” or “replanting,” the carcass with microbes carried in its gut. Several other explanations have been proposed for the application of exudates to the carcass, none of them mutually exclusive (reviewed in Trumbo, Sikes, & Philbrick, 2016). The hypothesis currently presumed by most studies is nevertheless that beetles use these fluids to defend the carcass from microbial competition through the “preserving” mechanism outlined above (Cotter et al., 2010; Rozen et al., 2008). Burying beetles also display behaviours that could prevent putrefaction, such as removal of the cadaver's intestine during carcass preparation (Eggert, Reinking, & Müller, 1998). Removing the intestines of the cadaver would likely prevent putrefaction caused by enteric microbes which would lead to rupture of the body cavity and promote microbial succession on the carcass (Metcalf et al., 2013). It also potentially provides a source of microbes for “replanting,” if the cadaver's intestines are consumed by beetles.

The effect of the beetles’ antimicrobial defences on the whole bacterial community on the carcass is unknown, and the structure of the bacterial community on the prepared carcass has never been described before. We combined detailed behavioural observations with quantitative real‐time PCR, next‐generation sequencing and bioinformatic analyses in an experimental approach to test our predictions, and deduce the mechanism(s) by which burying beetles may manipulate the bacterial community on their carcass breeding resource. To evaluate the “replanting” and “seeding” mechanisms in more detail, we also examined changes in the bacterial community within the burying beetle's gut and exudates during reproduction.

## 2 MATERIALS AND METHODS

### 2.1 Filming carcass preparation

We filmed carcass preparation of 20 pairs of burying beetles from a laboratory stock population maintained using a standard protocol (Cotter et al., 2010) under infra‐red light in the laboratory (details in Video S1), to determine whether removal of the gut is an integral and repeatable part of carcass preparation and therefore a potential source of microbes for “replanting.”

### 2.2 Bacterial communities on mouse carcasses: an experimental analysis

In September 2012, we collected *N. vespilloides* individuals from traps in Byron's Pool nature reserve in Grantchester, Cambridgeshire, UK (ordnance survey grid reference TL436546). Beetles were kept under standard laboratory conditions in individual boxes (12 × 8 × 2 cm) filled with moist garden compost and fed approximately 1 mg minced beef twice per week. To ensure all experimental individuals were sexually mature and had experienced similar conditions before the experiment, we allowed them to breed once under standard laboratory conditions (described in Cotter et al., 2010). After reproduction, adults were kept for use in the experiment described next.

The following week, we collected soil at six separate locations near our beetle traps in Byron's Pool. Some of the soil was placed in sterile sample bags and frozen at −80°C within 4 hr of collection for assessment of bacterial communities. The remaining soil was brought back to the laboratory. In one location (S3), we dug two separate holes; the two holes were sampled individually.

We filled 19 breeding boxes to half of their height using soil collected in the field. We placed a thawed mouse carcass (LiveFoods Direct^™^, previously kept at −20°C) on top of the soil in each box (day 0 of the experiment). These carcasses were then subjected to one of the following three treatments. On day 1, we sampled bacterial communities on six of the carcasses (hereafter called the “fresh” treatment). On the same day, we introduced a male–female pair of field‐caught *N. vespilloides* to seven other carcasses (“beetle” treatment). Burying beetles prefer breeding on fresh carcasses over decaying ones, potentially because decayed carcasses yield lower reproductive success (Rozen et al., 2008). Hence, our choice of introducing beetles after 1 day of decomposition reflects the species’ natural behaviour. The remaining six carcasses were manually buried (approximately 2 cm deep) in the soil to mimic the burial performed by beetles (“buried” treatment). Wearing gloves, we opened a small hole in the soil in the plastic box, and placed the carcass in it, covering it again loosely with soil. Importantly, the boxes of beetle‐prepared carcasses and buried carcasses were opened the same number of times, so exposure to airborne microbes was similar. On day 3, we removed beetle pairs from the beetle treatment because carcass preparation was complete. We identified the carcass as “prepared” when the fur was largely shaven, the surface showed signs of small incisions and being smeared with antimicrobial exudates (these have a dark brown‐dark red colour, thus darkening the flesh of the carcass and moistening it), and finally, rolled into a ball. The last step of carcass preparation (rolling into a ball) was not observed for all carcasses on day 3, which is fairly typical of the variation found in *N. vespilloides*. Broods thrive just as well in rounded and not‐rounded carcasses (De Gasperin, Duarte, Troscianko, & Kilner, 2016), hence rounding the carcass does not seem to be as important a step as the previous ones (shaving, incisions and smearing with exudates). We therefore removed all beetles on day 3, and maintained the same sample schedule for all carcasses. We allowed carcasses to rest for a day to minimize differences between carcasses simply due to the physical manipulation of carcasses by beetles. On day 4, we sampled bacterial communities on the beetle‐prepared and buried carcasses. None of the sampled carcasses showed signs of purging decomposition fluids or bloating, hence were not yet undergoing Active Decay (as defined in Megyesi, Nawrocki, & Haskell, 2005). Fresh, beetle‐prepared and buried carcasses were sampled following the same protocol. We first removed as much soil debris as possible with sterile tweezers. We rolled the carcass in 40 ml of sterile phosphate‐buffered saline (PBS) on Petri dishes, using a sterile swab to release as much material as possible from every region of the carcass into the PBS solution. We pipetted the solution into a 50‐ml tube and pelleted the bacterial cells and debris at 3930× *g* for 10 min. We discarded the supernatant and stored the pellet at −80°C until DNA extraction.

Analysing bacterial samples from fresh carcasses allowed us to characterize the bacterial communities present on the surface of the carcass before introduction of beetles. By comparing bacterial communities on the beetle‐prepared and buried carcasses, we could account for changes in microbial community that were due to carcass age and burial.

### 2.3 Bacterial communities in the burying beetle's gut and exudates

Next we analysed the bacterial communities associated with female burying beetles, to further investigate the “seeding” and “replanting” mechanisms. We focused on females because they remain longer with the brood than males (De Gasperin, Duarte, & Kilner, 2015; Scott, 1998), invest more than the male in antibacterial defences (Cotter & Kilner, 2010) and provide most of the direct care (Smiseth & Moore, 2004). We collected individuals from Byron's Pool in June 2013. Female beetles were kept under standard laboratory conditions and allowed to breed once with laboratory stock beetles, 1 week before the experiment took place. Hence, all females were sexually mature and had bred previously, just as in the experiment analysing the carcass bacterial communities.

After breeding, females were retained. Five of those females were placed with a virgin male from the laboratory stock population in breeding boxes half‐filled with moist compost and provided with a thawed mouse carcass. Three days later, at the time of larval hatching, we collected anal exudates from the breeding females, using a standard procedure (described in Cotter & Kilner, 2010). Exudates were collected with a capillary tube and diluted in 200 μl of sterile PBS. On the same day, we collected exudates of four non‐breeding females that had been kept in individual boxes, without access to a male or a carcass. Breeding and non‐breeding females were then anesthetized with CO_2_, surface‐sterilized with 96% ethanol and their entire gut was resected. Beetle guts were placed in centrifuge tubes with sterile PBS. Gut and exudate samples were stored at −80°C until DNA extraction.

### 2.4 Molecular analysis

DNA was isolated using the FastDNA^®^ Spin Kit for Soil (MP Bio Laboratories, Inc., Carlsbad, CA, USA) and stored at −20°C until use.

To compare bacterial abundance in the different carcass treatments, we performed quantitative real‐time polymerase chain reaction (qRT‐PCR) on a fragment of the 16S rRNA‐encoding gene (detailed methods in Data S1).

For library construction, we first PCR‐amplified the full‐length bacterial 16S rRNA‐encoding gene (using primer pair 27F/U1492R; Weisburg, Barns, Pelletier, & Lane, 1991). We confirmed the presence of the amplicon by agarose gel electrophoresis. The amplicon band was excised from the gel and the DNA was extracted with the Wizard^®^ SV Gel and PCR Clean‐Up System (Promega, Madison, WI, USA). DNA was quantified using a NanoDrop ND‐1000 Spectrophotometer.

To amplify the V3 region of the 16S rRNA‐encoding gene, a second PCR was run using 10 ng of amplicon template DNA per sample, with Illumina‐compatible primers and PCR conditions described in Bartram, Lynch, Stearns, Moreno‐Hagelsieb, and Neufeld (2011). Amplification of the V3 region was confirmed by agarose gel electrophoresis and DNA was extracted from the corresponding band (300 bp). High‐throughput paired‐end sequencing was carried out using an Illumina MiSeq instrument at the DNA Sequencing Facility (Department of Biochemistry, University of Cambridge). Sequence reads (NCBI BioProject PRJNA330954) were analysed using MOTHUR v.1.35.1 (www.mothur.org) software package (Schloss et al., 2009), following the Standard Operating Procedure described in Kozich, Westcott, Baxter, Highlander, and Schloss (2013) and MOTHUR's Wikipedia page (http://www.mothur.org/wiki/MiSeq_SOP, accessed August 2015). The quality filtering steps are described in detail in Data S1. Briefly, we trimmed sequences to reduce sequence variation due to sequencing errors and removed sequences with more than six homopolymers. We aligned sequences to the SILVA release 119 reference alignment and excluded those with low search scores and low similarity to the template sequences. Sequences were further de‐noised during pre‐clustering by clustering sequences with a difference of two or fewer nucleotides. Chimeric sequences were removed using UCHIME (Edgar, Haas, Clemente, Quince, & Knight, 2011). The remaining sequences were taxonomically classified by comparison against the SILVA release 119 reference database. Taxonomic assignment was made at each level, given a bootstrap value greater than 80, using the Ribosomal Database Project Classifier (Wang, Garrity, Tiedje, & Cole, 2007). Sequences classified as Chloroplast, Mitochondria, Archaea, Eukaryota or unknown at the kingdom level were removed. Uncorrected pairwise distances were calculated between sequence reads, using the DNADIST algorithm within MOTHUR with default options (full details in Data S1). Sequences at a distance threshold of 0.03 were then clustered into operational taxonomic units (OTUs), using the average neighbour algorithm (Schloss & Westcott, 2011). A consensus classification for each OTU was obtained. A data matrix was generated with every OTU and the number of reads belonging to each sample assigned to each OTU (available at the Cambridge Apollo repository: https://doi.org/10.17863/cam.9623). To control for differences in the number of reads obtained per sample, we used a subsample of the dataset in all analyses of 10760 (rarefaction curves in Figures S1 and S2). This was chosen because the smallest number of reads in any sample was 10760, obtained in a gut tissue sample of breeding beetles.

### 2.5 Statistical analysis

#### 2.5.1 Bacterial load

All statistical analyses were carried out in the statistical program R (R Core Team, 2016). Differences in bacterial DNA concentration between carcass treatments, estimated by qPCR, were tested with nonparametric statistics (Kruskal–Wallis test, and for post hoc comparisons, Dunn test) because the data were not normally distributed.

#### 2.5.2 Community richness and diversity

Differences in observed richness and diversity between carcass treatments (measured using the inverse Simpson index) were tested with ANOVA. For the analysis of community richness and diversity in beetle guts and exudates, we used sample type (gut or exudate) and breeding condition (breeding, non‐breeding) as factors in an ANOVA. The inverse Simpson index was log‐transformed for the beetle guts and exudates data because a Levene test indicated variance heterogeneity.

#### 2.5.3 Community membership

Non‐metric multidimensional scaling (NMDS) with three dimensions was applied to Bray–Curtis distance matrices to visualize distances between samples. Differences between communities were tested with PERMANOVA in R (vegan package, Oksanen et al., 2015). The same model structure as the ANOVAs described above was used for PERMANOVA. Multivariate group dispersions (variances) were calculated with the betadisper function (vegan package) and an ANOVA was performed to test for multivariate homogeneity of variances. A posteriori comparisons between levels of factors were obtained by customizing the model's contrast matrix (R script provided as Data S1).

To account for phylogenetic similarities between bacterial communities, we obtained uniFrac (weighted and unweighted) distance matrices from MOTHUR and tested for differences between communities based on uniFrac distances using PERMANOVA in R.

To identify OTUs strongly associated with each treatment and combinations of treatments, we used Indicator Species Analysis in R (De Cáceres & Legendre, 2009). This is a standard community ecology approach that takes into account both relative abundance and relative frequency of occurrence in various sites (Dufrêne & Legendre, 1997). The indicator value is highest when all occurrences of an OTU are found in a single group of sites (i.e. treatments) and when the OTU occurs in all instances of that group (i.e. samples within a treatment).

## 3 RESULTS

### 3.1 Carcass preparation behaviour

In all 20 filmed pairs, beetles were observed making an incision in the abdomen of the mouse carcass (average time for occurrence of incision: 7.6 hr after pairing). Eight of those pairs then buried the carcass, obscuring further observations. On the remaining 12 carcasses, nine had guts visibly hanging from the carcass (average time: 11.6 hr after pairing). It was not possible to visually confirm that beetles ate the guts because of continuing carcass preparation. However, the beetles manipulated the mouse's intestine with their mouthparts, which leads us to assume that some transfer of intestinal content occurred.

### 3.2 Bacterial communities on the carcass

#### 3.2.1 Bacterial load on the carcass

**Figure 1:**
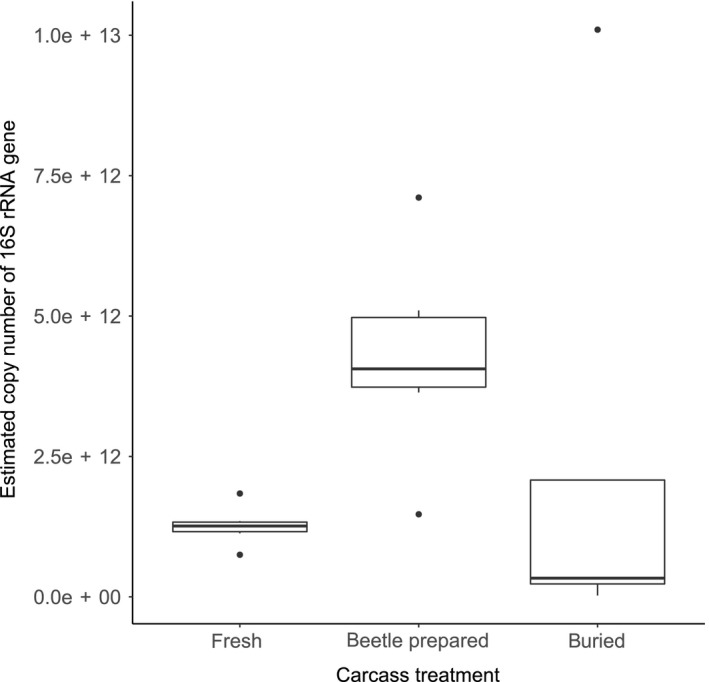
Estimated copy number of the 16S rRNA gene, quantified by quantitative real‐time polymerase chain reaction in the three carcass treatments: fresh, beetle‐prepared and buried

There was an overall effect of carcass treatment on bacterial load, estimated in copy numbers of the 16S rRNA gene (Kruskal–Wallis test: χ^2^ = 7.15, *p* = .03; Figure 1). Beetle‐prepared carcasses had significantly higher copy numbers of the 16S rRNA gene than fresh carcasses (Dunn test: *Z* = 2.37, Benjamini–Hochberg‐adjusted *p* = .026) and manually buried carcasses (*Z* = 2.15, Benjamini–Hochberg‐adjusted *p* = .024). Bacterial load did not differ significantly between fresh and buried carcasses (*Z* = 0.10, *p* = .46).

#### 3.2.2 Richness and diversity of bacterial communities

Across all samples we found that most OTUs occurred at very low sequence abundances. After subsampling, the OTU table for soil samples contained 2653 OTUs, with just 9% of those responsible for 80% of total sequence abundance. In carcass samples, after subsampling, 549 OTUs remained. Just seven of those OTUs (1.4% of the total) contributed to 80% of the total sequence abundance.

The highest OTU richness and diversity was observed in soil samples (Table 1). Observed richness was significantly higher in buried carcasses than in fresh and beetle‐prepared carcasses (Table 1; effect of treatment: *F*_2_ = 10.62, *p* = .001). Carcasses prepared by beetles and fresh carcasses showed similar levels of observed richness (Table 1; Tukey post hoc comparison: adjusted *p* = .76). Bacterial taxonomic diversity, assessed as the inverse Simpson index, also differed significantly between carcass treatments (effect of treatment: *F*_2_ = 5.93, *p* = .012). Buried carcasses showed the greatest diversity, while measures for beetle‐prepared and fresh carcasses were lower and similar to each other. Diversity was higher in buried than fresh (adjusted *p* = .012) and beetle‐prepared carcasses, although in the latter case, the difference was marginally non‐significant (adjusted *p* = .087). Fresh and beetle‐prepared carcasses showed similar levels of diversity (adjusted *p* = .451). Hence, despite the higher concentration of bacterial DNA in beetle‐prepared carcasses, these bacterial communities comprised fewer species than unprepared carcasses of the same age.

**Table 1:** Means and standard errors of observed richness and diversity (inverse Simpson) index for the different sample types

Type of sample	Observed richness	Inverse Simpson index
Soil	1078.95 ± 36.07	73.65 ± 7.24
Fresh carcasses	62.97 ± 5.32	1.76 ± 0.45
Beetle carcasses	50.83 ± 12.25	2.59 ± 0.40
Buried carcasses	126.48 ± 17.73	4.13 ± 0.60
Beetle guts—breeding	79.22 ± 6.74	3.39 ± 0.87
Beetle guts—non‐breeding	39.24 ± 4.69	1.16 ± 0.03
Beetle exudates—breeding	73.66 ± 4.64	3.43 ± 0.32
Beetle exudates—non‐breeding	65.39 ± 9.73	1.80 ± 0.36

#### 3.2.3 Community composition

**Figure 2:**
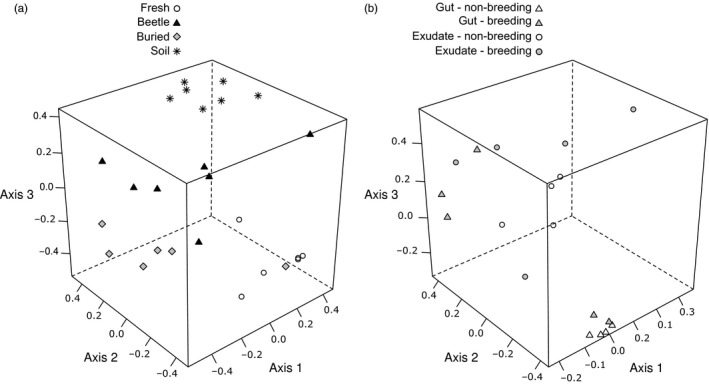
Non‐metric multidimensional scaling plot of the three dimensions of an ordination of (a) 2653 bacterial operational taxonomic units (OTUs) present in soil and carcass samples and (b) 284 bacterial OTUs in beetles’ gut and exudates

There were significant differences in bacterial community composition across all treatments. Soil communities differed significantly from carcass communities (Pseudo‐*F* = 16.183, *p* = .001; Figure 2). Communities on fresh carcasses were significantly different from communities on beetle‐prepared carcasses (Pseudo‐*F* = 10.217, *p* = .001). There were also significant differences between beetle‐prepared and buried carcasses (Pseudo‐*F* = 5.262, *p* = .002). Group dispersions were not significantly different between carcass treatments (*F* = 0.672, *p* = .524). PERMANOVA results on uniFrac distances were in agreement with results obtained with Bray–Curtis distances (Table S1).

**Figure 3:**
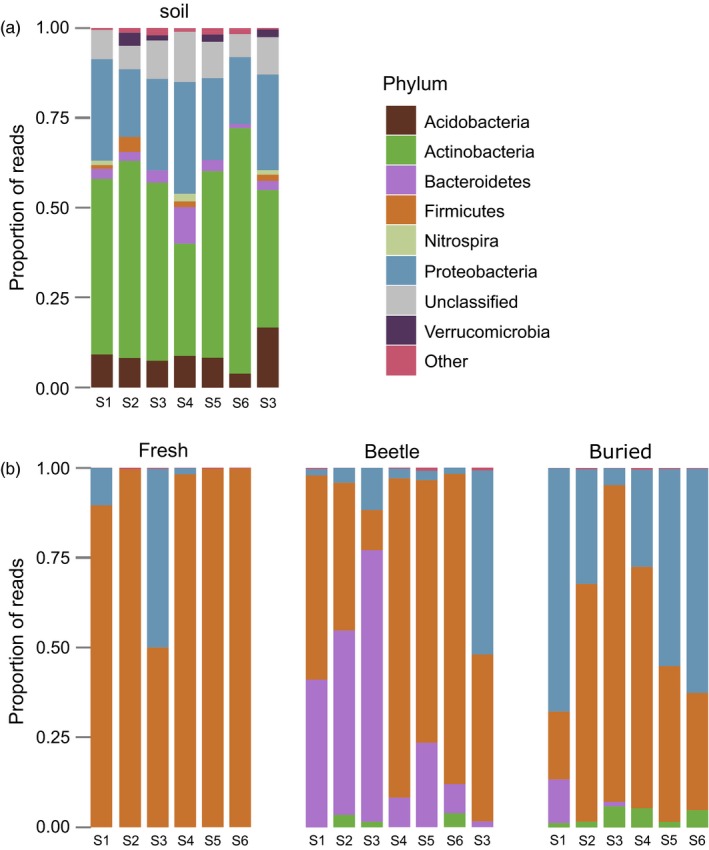
Relative abundance of major bacterial phyla within (a) soil samples and (b) carcass samples. Each bar represents a different sample. Labels below bars indicate the location from where soil was collected; in carcass samples, this was the soil used to fill breeding boxes

The most common bacterial phyla in soil communities were Actinobacteria and Proteobacteria (Figure 3a; see Figure S3a for visualization at the taxonomic level of order). Acidobacteria and Bacteroidetes were also common, yet contributed lower proportions of reads. There were also 472 OTUs that could not be classified to phylum level.

In fresh carcasses, the majority of reads belonged to Firmicutes OTUs (Figure 3b; see Figure S3b for visualization at the taxonomic level of order). In one sample, Proteobacteria OTUs composed approximately 50% of the observed reads, but this phylum was observed in low proportions in all other fresh carcass samples. Beetle‐prepared samples were mostly comprised of Firmicutes, Bacteroidetes and, in one sample, Proteobacteria. Manually buried carcasses were mostly dominated by Firmicutes and Proteobacteria, which is in agreement with previous characterizations of microbial communities in decomposing mouse cadavers (Metcalf et al., 2013).

### 3.3 Indicator analysis

To determine which groups are driving the differences in bacterial communities between carcass types, we used Indicator Species Analysis to identify OTUs significantly associated with different types of samples. Just three Bacillales OTUs were indicator species of fresh carcasses, the most abundant being *Bacillus*. For beetle‐prepared carcasses, the most abundant indicator OTUs were a Planococcaceae, two Flavobacteriaceae (one unclassified and one *Myroides*) and one Moraxellaceae (*Acinetobacter*) (Table 2, Figure 5). Other low abundance groups were also significantly associated with beetle‐prepared carcasses: one Microbacteriaceae, two Clostridiales (one unclassified and one *Tissierella*) and two Enterococcaceae (*Enterococcus* and *Vagococcus*). For buried carcasses, the most abundant indicator OTUs were a Planococcaceae (*Kurthia*) and a Psedomonadaceae (*Pseudomonas*). Present in lower abundances, one Micromonosporaceae, a Planococcaceae, one Enterobacteriaceae (*Escherichia–Shigella*), another *Pseudomonas* and one unclassified Alphaproteobacteria were also indicator species (Table 2, Figure 5).

**Table 2:** Bacterial taxa associated with different treatments using Indicator Species Analysis. Carcass samples were analysed separately from beetle‐originated samples (gut and exudate) to identify indicator groups for different carcass treatments. Gut and exudate samples were grouped by breeding status to identify indicator groups of breeding versus non‐breeding beetles. We report mean proportion of reads of indicator OTUs in the treatments of which they are indicators. Proportions were averaged over OTUs when multiple OTUs exist under the same classification. Only significant (*p* < .05) taxa with indicator value (IV) >0.85 are shown

Treatment	Order	Family	Genus	OTU ID	Mean proportion reads	IV
Fresh carcasses	Bacillales	Bacillaceae_1	*Bacillus*	1	0.681	0.857
	Bacillales	Unclassified	Unclassified	18,49	0.010	0.874
Beetle carcasses	Actinomycetales	Microbacteriaceae	Unclassified	20	0.011	0.917
	Bacillales	Planococcaceae	Unclassified	3	0.407	0.932
	Clostridiales	Clostridiales_Incertae_Sedis_XI	*Tissierella*	11	0.002	0.996
	Clostridiales	Unclassified	Unclassified	12	0.067	0.998
	Flavobacteriales	Flavobacteriaceae	*Myroides*	2	0.263	0.996
	Flavobacteriales	Flavobacteriaceae	Unclassified	28	0.012	0.925
	Lactobacillales	Enterococcaceae	*Enterococcus*	17	0.001	0.851
	Lactobacillales	Enterococcaceae	*Vagococcus*	6	0.011	0.862
	Pseudomonadales	Moraxellaceae	*Acinetobacter*	7	0.095	0.995
Buried carcasses	Actinomycetales	Micromonosporaceae	Unclassified	1	0.002	0.951
	Bacillales	Planococcaceae	*Kurthia*	4	0.157	0.916
	Bacillales	Planococcaceae	*Lysinibacillus*	36	0.005	0.882
	Enterobacteriales	Enterobacteriaceae	*Escherichia_Shigella*	62	0.004	0.872
	Pseudomonadales	Pseudomonadaceae	*Pseudomonas*	8,13,23	0.110	0.977
	Pseudomonadales	Pseudomonadaceae	Unclassified	179	0.001	0.873
	Alphaproteobacteria	Unclassified	Unclassified	24,43	0.0001	0.868
Breeding beetles	Bacillales	Planococcaceae	*Kurthia*	4	0.016	0.976
	Bacillales	Planococcaceae	Unclassified	3	0.127	0.996
	Clostridiales	Unclassified	Unclassified	12	0.053	0.992
	Enterobacteriales	Enterobacteriaceae	*Providencia*	16	0.002	0.894
	Enterobacteriales	Enterobacteriaceae	Unclassified	29,39,54	0.007	0.947
	Flavobacteriales	Flavobacteriaceae	*Myroides*	2,9	0.069	0.998
	Lactobacillales	Streptococcaceae	*Lactococcus*	153	0.003	0.894
	Pseudomonadales	Moraxellaceae	*Acinetobacter*	7	0.015	0.994
	Pseudomonadales	Moraxellaceae	Unclassified	41	0.001	0.885
	Xanthomonadales	Xanthomonadaceae	*Wohlfahrtiimonas*	5	0.012	0.978
Non‐breeding beetles	Bacillales	Unclassified	Unclassified	49	0.0007	0.883
	Clostridiales	Ruminococcaceae	Unclassified	270	0.002	0.906

As Figure 5 illustrates, few OTUs were exclusively present in beetle‐prepared carcasses. *Myroides*,* Vagococcus* and Planococcaceae, for example, show the highest abundances in beetle‐prepared carcasses, and are also present at low abundances in buried carcasses. By contrast, Clostridiales were only found in beetle‐prepared carcasses and beetle tissues (see below). The *Acinetobacter* OTU which is an indicator of beetle‐prepared carcasses was not found in either fresh or buried carcasses, although other *Acinetobacter* OTUs were. Further sequencing of different regions of the 16S rRNA gene is required to understand the degree of differentiation between these *Acinetobacter* OTUs.

### 3.4 Bacterial communities in *N. vespilloides* guts and exudates

#### 3.4.1 Richness and diversity of bacterial communities

Most OTUs occurred at very low sequence abundances: 284 OTUs remained after subsampling, of which just 4 (1.4% of the total) contributed to more than 80% of total sequence abundance. These OTUs were classified as *Bacillus* sp. (36.6% reads), *Vagococcus* sp. (30%), *Myroides* sp. (7.4%) and an unclassified *Planococcus* OTU (7.1%). OTU richness was overall higher in breeding beetles than in non‐breeding beetles (Table 1; *F* = 13.22, *p* = .003). The lowest observed richness was found in guts of non‐breeding beetles. We found a significant interaction between breeding status and type of sample (gut or exudate), largely driven by a difference in richness between guts and exudates of non‐breeding beetles, although statistically this difference was marginally non‐significant (*p* = .080). Breeding beetles showed higher diversity than non‐breeding beetles (*F* = 12.25, *p* = .003), independent of the type of sample (guts or exudates).

#### 3.4.2 Community composition

**Figure 4:**
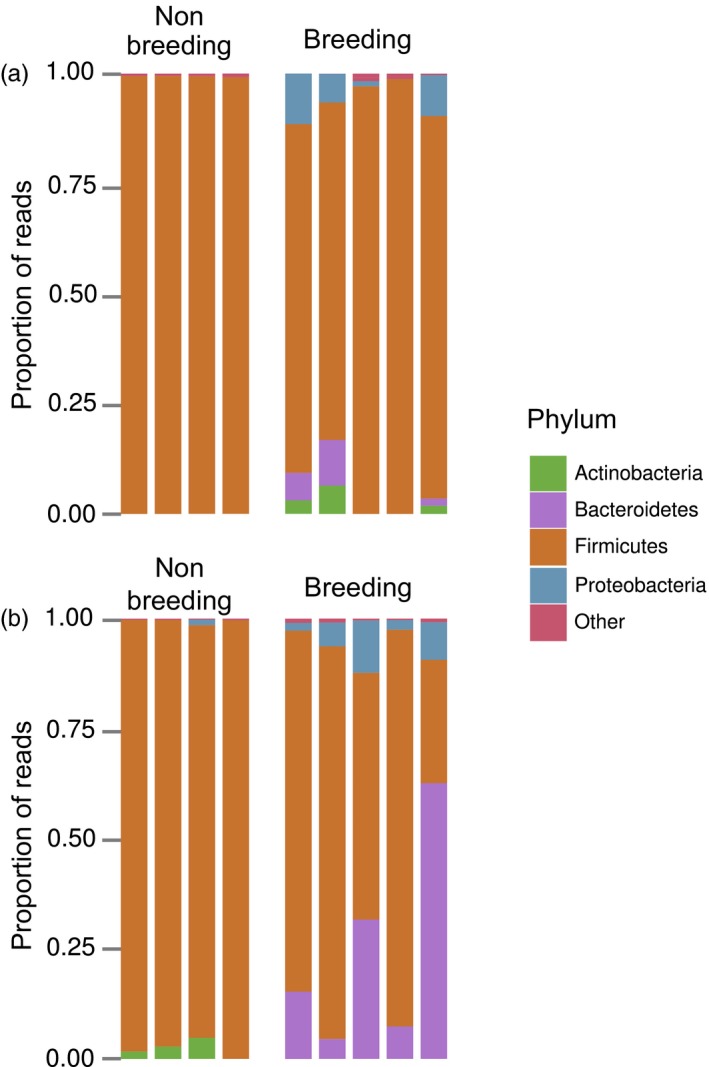
Relative abundance of major bacterial phyla within (a) gut of non‐breeding and breeding beetles, and (b) exudates of non‐breeding and breeding beetles. Each bar corresponds to a sample taken from one individual. Individuals are presented in the same order for gut and exudate samples, such that samples belonging to the same individual are vertically aligned

Beetle breeding status had a significant effect on the composition of bacterial communities (Pseudo‐*F* = 5.189, *P*(Perm) = 0.016), which suggests that the changes in bacterial communities that arise when beetles are breeding are temporary and environmentally induced. We also found an interaction between breeding status and type of sample (*P*(Perm) = 0.010). To further investigate this, we customized model contrasts for post hoc testing. The model with customized contrasts revealed that differences between bacterial communities in the guts and exudates were marginally non‐significant in breeding beetles (*P*(Perm) = 0.086), but significantly different in non‐breeding beetles (Pseudo‐*F* = 10.771, *P*(Perm) = 0.001; Figure 4; see Figure S4 for visualization at the taxonomic level of order). Multivariate group dispersions (variances) were significantly different between treatments (*F* = 7.633, *p* = .003). In particular, guts and exudates of non‐breeding beetles showed low multivariate dispersions (average distance to median = 0.03 and 0.16, respectively), while guts and exudates of breeding beetles showed high multivariate dispersions (average distance to median = 0.45 and 0.37, respectively). Therefore, PERMANOVA results should be interpreted with caution (Anderson, 2001). In conjunction with the NMDS plot (Figure 2b), a conservative interpretation would be that community composition is much more variable in breeding beetles than in non‐breeding beetles.

Firmicutes were the most common group in all samples (Figure 4). There were similarities between beetle‐prepared carcasses and samples from breeding beetles: particularly, exudates from breeding beetles showed a high proportion of Bacteroidetes (Figure 4b), just as in beetle‐prepared carcasses. Proteobacteria and Actinobacteria also comprised a small proportion of reads in breeding beetles.

### 3.5 Indicator analysis

**Figure 5:**
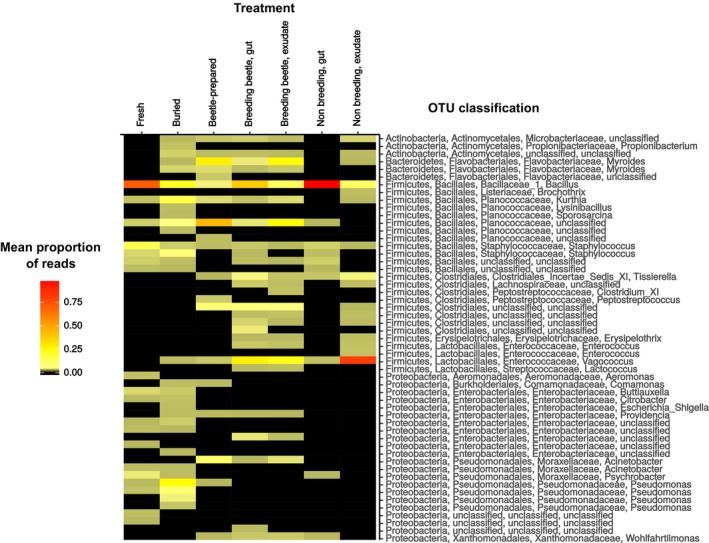
Mean proportion of reads assigned to each operational taxonomic unit (OTU) across different carcass and beetle treatments. Each row corresponds to a different OTU, classified to genus level whenever possible. OTUs with the same classification were not pooled because different OTUs may show different patterns of abundance across treatments. Mean proportions between 0% and 1% were depicted in black, for the remaining values a colour scale was used (see legend) with light yellow indicating low proportions and red indicating high proportions

We searched for indicator species that distinguish bacterial communities in breeding beetles from those in non‐breeding beetles, combining samples (guts and exudates) within those categories. Breeding beetles shared four indicator OTUs with beetle‐prepared carcasses: one Flavobacteriaceae (*Myroides*), one unclassified Planococcaceae, one Moraxellaceae (*Acinetobacter*) and one unclassified Clostridiales OTU. These OTUs, which were the most abundant indicator OTUs in beetle‐prepared carcasses, were also abundant indicator OTUs in breeding beetles (Figure 5). Breeding beetles shared a single Planococcaceae OTU (*Kurthia*) with buried carcasses. Furthermore, four Enterobacteriaceae, one Streptococcaceae (*Lactoccocus*), one unclassified Moraxellaceae and one Xanthomonadaceae (*Wohlfartiimonas*) OTU were indicators of breeding beetles, in comparison to non‐breeding beetles. One unclassified Bacillales and one Ruminococcaceae (Clostridiales) OTU were the only indicators of communities of non‐breeding individuals (Table 2).

The breeding beetle indicator Xanthomonadaceae OTU (classified as *Wohlfartiimonas*) was also present, at lower abundances, in beetle‐prepared carcasses and guts of non‐breeding beetles (Figure 5). Some Clostridiales, such as *Tissierella* were present in almost all beetle samples, as well as beetle‐prepared carcasses. Other Clostridiales were only found in beetle guts or exudates, although many of them were absent from non‐breeding beetle's guts.

## 4 DISCUSSION

We propose four potential mechanisms by which animals might actively decrease competition from microbes for a key resource, and which are not mutually exclusive: preserving, weeding, replanting and seeding. We deduced which of these mechanisms accounts for the way in which burying beetles manage the bacterial community on their carcass breeding resource.

### 4.1 Preserving

Our results suggest that the current assumption that burying beetles “preserve” the carcass (e.g. Rozen et al., 2008) is not valid, because we found that beetles increase the bacterial load on their breeding resource. These findings are consistent with the conclusions of a recent behavioural study suggesting that other *Nicrophorus* species do not eliminate the microbial community on a carcass either (Trumbo et al., 2016). It is possible that the increased bacterial load observed in beetle‐prepared carcasses, when compared with fresh or buried carcasses, may be due to bacterial growth occurring during the 24 hr between beetle removal and carcass sampling. However, buried and beetle‐prepared carcasses were sampled at the same time, hence had the same time for bacterial growth to occur. At the very least, our results show that beetle‐prepared carcasses offer an environment which is highly amenable for bacterial growth, contrary to that previously assumed.

Manually buried carcasses showed surprisingly low levels of bacterial load, which suggests that, at least on the external surface of the carcass, microbes are not a major source of competition. It is possible that microbial loads were higher inside the carcass, where decomposition starts. We sampled the external surface of the carcass because this is what burying beetles work on during carcass preparation, apart from the removal of intestines. Our results are nevertheless congruent with time‐course studies on mouse cadaver decomposition, where signs of bloating and purging (early stages of decay) only appear 6 days postmortem, with accompanying changes in the internal microbial community (Metcalf et al., 2013). Possibly, the most effective behaviour shown by beetles to prevent active decay is the removal of the cadaver's gut. The preparation of the external surface of the carcass by shaving and applying exudates may suit other purposes, such as reducing odour cues for other beetles (Suzuki, 1999), preventing carcass desiccation or attracting larvae to the carcass (Pukowski, 1933).

### 4.2 Weeding

Despite the increased bacterial load, we found that bacterial communities on beetle‐prepared carcasses showed levels of species richness and diversity that were no different from those associated with a fresh carcass. Yet results from the manually buried carcasses suggest that burial of the carcass alone is sufficient to increase levels of species richness and diversity—probably due to colonization by the highly rich and diverse soil bacteria (Figure 2a). Perhaps, the antimicrobial substances produced by beetles restrict the diversity of species that can grow on a carcass after its burial by beetles. In short, by burying a carcass to protect it from rival animals, beetles expose it to a new set of rival bacteria, which are then eliminated, possibly by “weeding.” Another possible explanation is that a handful of fast‐growing bacteria out‐compete other groups in beetle‐prepared carcasses, which results in lower community diversity. An example of such a fast‐growing bacterium could be *Myroides*, which thrives in beetle‐prepared carcasses, but is scarce in manually buried carcasses.

Further evidence consistent with the “weeding” mechanism comes from our finding that bacterial community membership differed between beetle‐prepared carcasses and both fresh and manually buried carcasses (Figure 3b). In part, this was due to the reduction of some groups of bacteria, including some Gram negative bacteria as well as the Gram positive (such as Firmicutes) we expected to be removed by the lysozymes in the burying beetle's exudates (Jacobs et al., 2016; Palmer et al., 2016). The Gram negative Proteobacteria in particular were less abundant in beetle‐prepared carcasses than on the manually buried carcasses. The Proteobacteria include several insect pathogens, such as *Serratia*,* Enterobacter, Pseudomonas* and *Escherichia*–*Shigella* sp. (Bulla, Rhodes, & St. Julian, 1975), the latter two being indicator groups of manually buried carcasses. Hence, there is potentially strong selective pressure for beetles to reduce the abundance of these bacterial groups on their breeding resource.

### 4.3 Seeding and replanting

Consistent with the “seeding” and “replanting” mechanisms, bacterial communities on burying beetle‐prepared carcasses were changed by the addition of groups such as Clostridiales (*Tissierella* and other unclassified OTUs), Moraxellaceae (*Acinetobacter*) and Xanthomonadales (*Wohlfartiimonas*), the latter at very low abundances (Figure 5). These groups were found exclusively in beetle‐associated samples and are therefore likely candidates for replanting or seeding mechanisms. Interestingly, taxonomically similar groups were found across several carrion‐feeding beetles in the family Silphidae (Kaltenpoth & Steiger, 2014), which suggests an association between these bacteria and the type of resource utilized by carrion beetles. *Tissierella* is also present in the gut and exudate of non‐breeding beetles, which suggests a “seeding” mechanism, although other Clostridiales are absent from the non‐breeding beetles’ gut. We cannot completely rule out “replanting,” as the Clostridiales are common bacteria in soil and in the mammalian gut (Madigan, Clark, Stahl, & Martinko, 2010). Our behavioural observations demonstrate that removal and presumable consumption of the cadaver's intestine is an integral and repeatable part of carcass preparation by *N. vespilloides*. The presence of Clostridiales on the outside of the carcass could therefore result from “replanting” the mouse gut microbiota on the carcass via beetle oral and anal exudates. *Acinetobacter* are also common soil bacteria and could be “replanted” on the carcass from the surrounding soil. The absence of this bacterium from non‐breeding beetles suggests that the association between *Acinetobacter* and burying beetles is transient. *Wohlfartiimonas,* on the other hand, has only been found in the gut of insects (Kaltenpoth & Steiger, 2014; Lee et al., 2014; Tóth et al., 2008). In our study, this bacterium was only present in beetle‐associated samples, making it a likely candidate for “seeding,” despite its low abundance in beetle‐prepared carcasses. Note, however, that we cannot yet draw any conclusions about the adaptive value for burying beetles of the additions of the above‐mentioned groups to the carcass bacterial community. Such changes could simply be a by‐product of carcass preparation, and have no impact on beetle fitness.

To distinguish between “seeding” and “replanting,” further experiments are necessary where the microbiota of the breeding resource and/or surrounding soil are manipulated. An examination of bacterial communities on carcasses under different manipulations should provide interesting information regarding which groups are endogenous and “seeded” and which are environmentally acquired and “replanted.”

At the community level, breeding beetles differed from (non‐virgin) non‐breeding beetles (Figure 4), potentially because bacterial species richness and diversity was lower in the guts of non‐breeding females than the guts of breeding females. This is not surprising given that breeding beetles were exposed to more sources of bacteria than non‐breeding beetles, namely the carcass and a male beetle. Furthermore, some aspects of the internal immune system are down‐regulated during breeding (Cotter, Littlefair, Grantham, & Kilner, 2013; Jacobs et al., 2016; Reavey, Warnock, Vogel, & Cotter, 2014), which could allow more bacteria from the environment to colonize the gut. In breeding beetles, bacterial communities in guts and exudates do not differ statistically, potentially because of the contact with bacteria from multiple sources.

We did observe a significant difference between the communities in the gut and exudates of non‐breeding beetles but this could be due to the sampling method for the exudates, which were collected first. We were only able to surface sterilize beetles after exudate collection, before gut dissection, because burying beetles often release exudates upon being touched and then may not do so again for several hours. Therefore, it is possible that the exudates were contaminated with external bacteria, whereas the gut samples were not.

The bacterial communities of breeding beetles suggest a mix of environmentally acquired and endogenous microbiota. Interestingly, while adults appear to lose some members of their gut bacterial community after breeding, it has recently been shown that transfer of bacteria occurs between parents and offspring during the breeding event (Wang & Rozen, 2017). Therefore, while, within individuals, some beetle‐bacteria associations may be transient, there is nevertheless potential for vertical transmission and long‐term evolutionary associations with microbes in this species.

## 5 CONCLUSIONS

Burying beetles use a combination of behavioural and chemical strategies which change microbial communities on their breeding resource. Drawing on a wide variety of evidence, we refute the assumption that beetles “preserve” the carcass by reducing bacterial growth. Our data suggest that some bacterial groups are excluded from the carcass (“weeding”), but others thrive. A combination of “seeding” and “replanting” may occur in a few bacterial groups. It will be important in the future to also investigate whether similar shifts occur in fungal communities in the presence of burying beetles, and the role of known fungal endosymbionts (Kaltenpoth & Steiger, 2014) in the utilization of the breeding resource. The challenges for future work are to investigate the functionality of the microbial community that remains associated with breeding beetles on the carcass. While some groups may compete with burying beetles for resource utilization, others may be entomopathogens, and others may be beneficial because they help digesting carrion or produce antimicrobial substances themselves. More detailed studies will help determine whether the shift in the bacterial community that we have described is adaptive for burying beetles or simply a by‐product of carcass preparation.

## AUTHORS’ CONTRIBUTIONS

A.D., M.W. and R.M.K. conceived the ideas and designed methodology. A.D. and C.S. collected the data. A.D. and J.W. analysed the data. A.D. and R.M.K. led the writing of the manuscript. All authors contributed to the drafts and gave final approval for publication.

## DATA ACCESSIBILITY

Raw sequence reads are available in the NCBI Sequence Read Archive: BioProject PRJNA330954. Quantitative PCR data and OTU table available from Cambridge Apollo repository https://doi.org/10.17863/cam.9623 (Duarte, Welch, Swannack, Wagner, & Kilner, 2017).
