# Supplementary material for: Strategies for managing rival bacterial communities: Lessons from burying beetles
Source: J Anim Ecol. 2017 Aug 21;87(2):414–27. doi: 10.1111/1365-2656.12725 (PMC5836980; doi:10.1111/1365-2656.12725)
Supplement: Supplementary file 2 [file JANE-87-414-s002.docx]

**Strategies for managing rival bacterial communities: lessons from burying beetles**

**Ana Duarte, Martin Welch, Chris Swannack, Josef Wagner and Rebecca M. Kilner**

**Supplementary Methods**

**Video recordings of carcass preparation by burying beetles**

We put a male and female burying beetle in a 5L aspirator bottle with an Infrared Venus 2.0 USB camera placed above in the neck of the bottle. The bottle and camera were then placed in a dark, soundproof chamber. Video frames were recorded at 1 per second with a resolution of 640×480 using commercially available video capture software VideoVelocity (<http://www.candylabs.com/videovelocity>). The Venus 2.0 USB camera plugged directly into the laptop using the USB port.

### Quantitative real-time PCR

We chose a set of universal primers for the 16S rRNA gene, which amplify a 180 bp sequence within a conserved region of the gene. The primers have been reported to target 93.6% of all bacterial 16S rRNA sequences published and to be capable of detecting fewer than 100 copy numbers of the 16S gene (Clifford et al., 2012).

A standard dilution series was created using genomic DNA from *Pseudomonas aeruginosa*. DNA was quantified with Qubit Fluorometer. DNA concentration of the standard solutions ranged from 14.2 ng/µl to 1.42 x10^-5^ ng/ µl.

We assessed via standard PCR which dilution of the samples’ DNA to use for quantitative PCR. Samples amplified successfully either with 10-fold or 100-fold dilutions. In each well of a 96-well plate, 2.5 µl of template were added to 10 µl of Fast SYBR® Green Master Mix, 1.25 µl of both primers (10 umol), and 5 µl of Milli-Q ultrapure filter-sterilized water, resulting in a final reaction volume of 20 µl. Quantitative PCR was performed with the 7300 Real-Time PCR System from Applied Biosystems®. The samples, standard curve, a positive control of unquantified *P. aeruginosa* DNA and a no-template control were all run in triplicate in the same 96-well plate. We followed cycle conditions advised for Fast SYBR® Green Master Mix: 95 °C for 20 seconds, then 40 cycles of 95 °C for 3 seconds and 60 °C for 30 seconds.

### Estimating copy number of 16S rRNA gene concentration

There is a linear relationship between the threshold cycle (C_T_) and the log concentration of DNA in the template. From the standard dilution series of known DNA quantities we calculated the slope and intercept of this linear equation (fig. 1). We then estimated the concentration of bacterial DNA present in samples, based on average C_T_ values obtained for each sample, and multiplying by the dilution factors used. We then calculated copy numbers of the 16S rRNA gene in the templates using the following formulas (Clifford et al., 2012):

Molecular weight of amplicon in g/mol = Size of amplicon (in bp) × 650 Daltons/bp

# copies of amplicon in 1 ng of DNA = (1×10^−9^ g ÷ Mw of genome)×6.02×10^23^ molecules/mole (Avogadro’s number).

# copies in template = Concentration (ng/ul) of template × volume template (ul) × # copies of amplicon in 1 ng of DNA

**Quality filtering of Illumina sequences**

Sequences containing > 250bp and more than 6 homopolymers were removed. To reduce sequence variation introduced by sequencing errors, sequences were trimmed with PRINSEQ-lite v.0.20.3 by 20 bp at the beginning of the forward reads and by 10 bp at the end of the reverse reads. This was necessary due to poor sequence quality at the end of the reads. The remaining sequences were aligned to the SILVA release 119 reference alignment, trimmed to the V3 region of the 16S rRNA-encoding gene. A post alignment screening was conducted and sequences with a search score below 80% and a similarity to the template sequences below 90% were excluded. Sequences were further de-noised during the pre-clustering steps and sequences with a difference of just 2 nucleotides were clustered together. Chimeric sequences were removed using the UCHIME algorithm (Edgar, Haas, Clemente, Quince, & Knight, 2011) within MOTHUR. Sequences represented by fewer than 10 copies were excluded from further analysis. Those sequences were most likely left over low quality sequences escaping the quality filtering.

**OTU clustering**

Pairwise distances for OTU clustering in MOTHUR are calculated using an implementation of DNADIST (Felsenstein 1989), which compares two sequences and counts differences (mismatches) and gaps. It then calculates what percentage of nucleotides is different in the sequence. A distance of 0.03 means that sequences are 97% similar. The default option in MOTHUR is to treat strings of gaps as a single character (see https://www.mothur.org/wiki/Dist.seqs). Hence a sequence with 2 mismatched nucleotides and three consecutive gaps (a string of gaps) when compared to another sequence of the same length, would be considered to have only 3 differences.

**References**

Clifford, R. J., Milillo, M., Prestwood, J., Quintero, R., Zurawski, D. V, Kwak, Y. I., … Mc Gann, P. (2012). Detection of Bacterial 16S rRNA and Identification of Four Clinically Important Bacteria by Real-Time PCR. *PLoS ONE*, *7*(11), 1–6. https://doi.org/10.1371/journal.pone.0048558

Edgar, R. C., Haas, B. J., Clemente, J. C., Quince, C., & Knight, R. (2011). UCHIME improves sensitivity and speed of chimera detection. *Bioinformatics*, *27*(16), 2194–2200. https://doi.org/10.1093/bioinformatics/btr381

Felsenstein, J. 1989. PHYLIP—Phylogeny Inference Package. Cladistics 5:164–166.
