# Supplementary material for: Strategies for managing rival bacterial communities: Lessons from burying beetles
Source: J Anim Ecol. 2017 Aug 21;87(2):414–27. doi: 10.1111/1365-2656.12725 (PMC5836980; doi:10.1111/1365-2656.12725)
Supplement: Supplementary file 3 [file JANE-87-414-s003.docx]

# **Strategies for managing rival bacterial communities: lessons from burying beetles**

**Ana Duarte, Martin Welch, Chris Swannack, Josef Wagner and Rebecca M. Kilner**

**Supplementary Tables and Figures**

**Table S1.** Summary of PERMANOVA for uniFrac distances in carcass communities. First we performed a global test (i.e. overall effect of treatment), and if significant we used customized contrasts to compare groups.

| **Unweighed uniFrac** | **Pseudo-*F*** | ***R*^2^** | ***p*-value** |
| --- | --- | --- | --- |
| **Global test: Treatment** | 2.46 | 0.24 | 0.001 ** |
| **Fresh vs Beetle-prepared** | 2.67 | 0.13 | 0.001 ** |
| **Fresh vs Buried** | 2.25 | 0.11 | 0.002 ** |
| **Weighed uniFrac** |  |  |  |
| **Global test: Treatment** | 5.49 | 0.41 | 0.001 ** |
| **Fresh vs Beetle-prepared** | 6.72 | 0.25 | 0.001 ** |
| **Fresh vs Buried** | 4.26 | 0.16 | 0.002 ** |

**Figure S1.** Rarefaction curves for sequence data from carcass samples. The vertical line shows the value by which data was rarefied (subsampled). Horizontal lines show where this value intersects the rarefaction curves. A) All sequences included. B) Rare sequences excluded: only sequences with more than 10 copies in each sample were included.
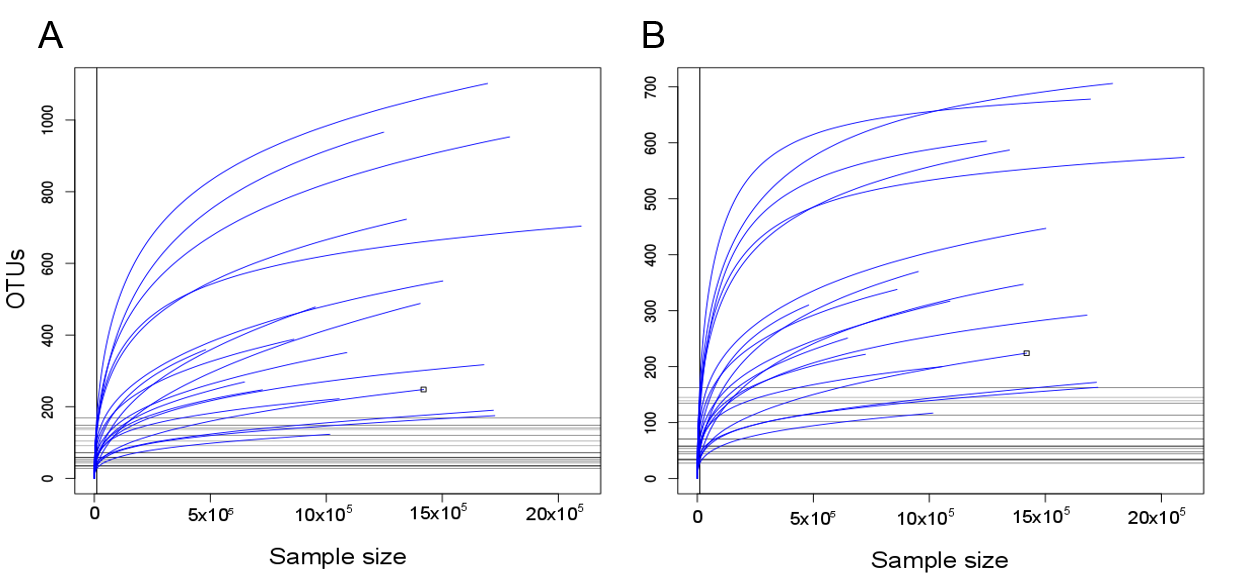


**Figure S2.** Rarefaction curves for sequence data from beetle samples. The vertical line shows the value by which data was rarefied (subsampled). Horizontal lines show where this value intersects the rarefaction curves. A) All sequences included. B) Rare sequences excluded: only sequences with more than 10 copies in each sample were included.


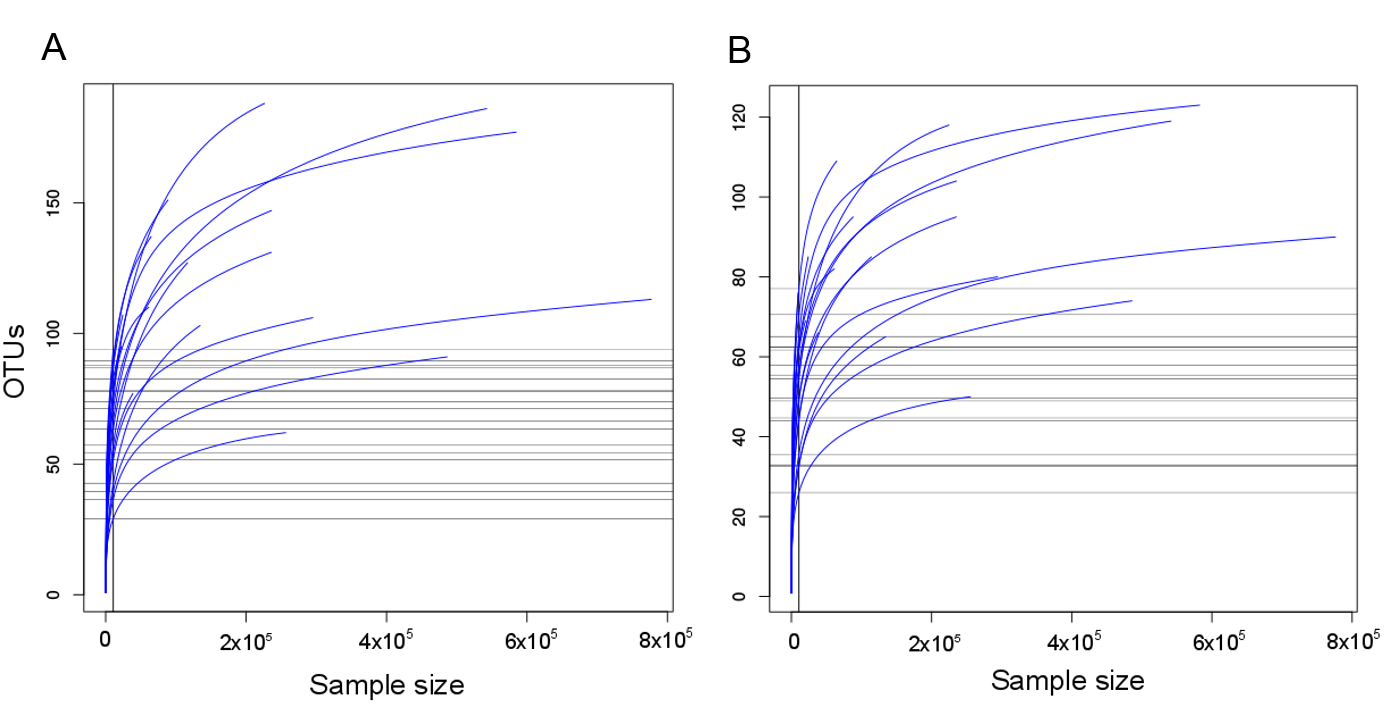


**Figure S3.** Relative abundance of major bacterial taxa classified at the Order level within **A**) soil samples and **B**) carcass samples. Each bar represents a different sample. Labels below bars indicate the location from where soil was collected; in carcass samples, this was the soil used to fill breeding boxes.

**
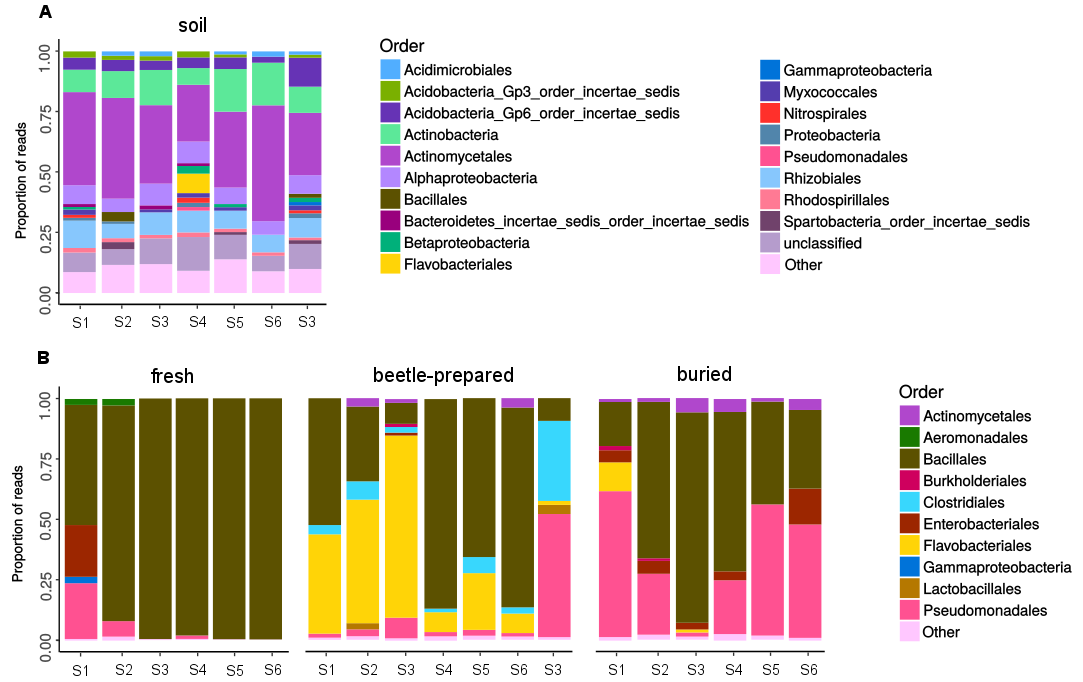
**

**Figure S4.** Relative abundance of major bacterial taxa classified at the Order level within **A**) gut of non-breeding and breeding beetles, and **B**) exudates of non-breeding and breeding beetles. Each bar corresponds to a sample taken from one individual. Individuals are presented in the same order for gut and exudate samples, such that samples belonging to the same individual are vertically aligned.

**
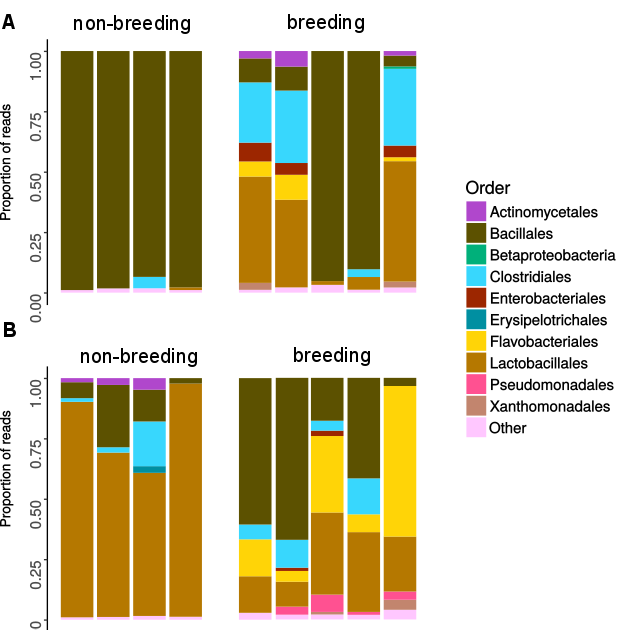
**
